# Ongoing Genome Reduction in *Mycobacterium ulcerans*

**DOI:** 10.3201/eid1307.060205

**Published:** 2007-07

**Authors:** Simona Rondini, Michael Käser, Timothy Stinear, Michel Tessier, Cyrill Mangold, Gregor Dernick, Martin Naegeli, Françoise Portaels, Ulrich Certa, Gerd Pluschke

**Affiliations:** * Swiss Tropical Institute, Basel, Switzerland; † Institut Pasteur, Paris, France; ‡ F. Hoffmann-La Roche Ltd., Basel, Switzerland; § Institute of Tropical Medicine, Antwerp, Belgium; 1 These authors contributed equally to this work.; 2 Current affiliation: Monash University, Melbourne, Victoria, Australia

**Keywords:** Mycobacterium ulcerans, deletional genomic diversity, microarray, strain variation, research

## Abstract

Elucidation of the transmission, epidemiology, and evolution of *Mycobacterium ulcerans*, the causative agent of Buruli ulcer, is hampered by the striking lack of genetic diversity of this emerging pathogen. However, by using a prototype plasmid-based microarray that covered 10% of the genome, we found multiple genomic DNA deletions among 30 *M. ulcerans* clinical isolates of diverse geographic origins. Many of the changes appear to have been mediated by insertion sequence (IS) elements IS*2404* and IS*2606*, which have high copy numbers. Classification of the deleted genes according to their biological functions supports the hypothesis that *M. ulcerans* has recently evolved from the generalist environmental *M. marinum* to become a niche-adapted specialist. The substantial genomic diversity, along with a prototype microarray that covered a small portion of the genome, suggests that a genome-wide microarray will make available a genetic fingerprinting method with the high resolution required for microepidemiologic studies.

## Abstract 2

*M. ulcerans* is adapting to a more stable environment.

The study of genetic diversity within bacterial species has provided information on aspects such as virulence (*1*,*2*), antimicrobial drug resistance (*3*), epidemiology, and microbial evolution (*4*–*7*). For mycobacteria such as *Mycobacterium tuberculosis* and *M. ulcerans*, low intraspecies diversity limits the use of genetic fingerprinting techniques that are based on sequence diversity in selected genetic elements. For *M. tuberculosis*, *M. bovis*, and the various bacillus Calmette-Guérin daughter strains, genome-wide microarray analyses have identified large sequence polymorphisms (*4*,*8*–*10*). However, the complete genome sequence of an organism is required for the design of synthetic oligonucleotide or PCR product–based microarrays. When this information is not available, an alternative is a PCR product–based shotgun DNA microarray (*11*), which we developed further into a plasmid-based microarray. We used this method for the differential genomic analysis of *M. ulcerans,* a human pathogen for which the fully assembled and annotated genome sequence was not available at the time of the study.

*M. ulcerans* is the causative agent of Buruli ulcer, an infectious disease characterized by chronic necrotizing skin ulcers (*12*). Buruli ulcer is an emerging infectious disease found mostly in West African countries but also in tropical and subtropical regions of Asia, the Western Pacific, and Latin America (*13*). Genetic analyses suggest recent divergence of *M. ulcerans* from *M. marinum*, a well-known fish pathogen that can cause limited granulomatous skin infections in humans (*14*). One of the hallmarks of the emergence of *M. ulcerans* as a more severe pathogen is the acquisition of a 174-kb plasmid that bears a cluster of genes necessary for the synthesis of the polyketide toxin mycolactone. This toxin appears largely responsible for the massive tissue destruction seen in Buruli ulcer (*15*). The epidemiology and mode of transmission of *M. ulcerans* disease are not fully understood, partly because no molecular typing method with sufficiently high resolution for microepidemiologic analyses is available.

Standard molecular typing methods such as multilocus sequence typing, restriction fragment length polymorphism, and fingerprinting using variable number of tandem repeats have shown an apparent lack of genetic diversity of *M. ulcerans* within individual geographic regions, which is indicative of a clonal population structure. The genotyping technique that has shown the highest discriminatory power so far is based on the use of outward-directed primers specific for the insertion sequence (IS) IS*2404*, in combination with an oligonucleotide that targets a repeated GC-rich motif (*16*). Application of this method determined the resolution of 10 different *M. ulcerans* genotypes, which correspond to the geographic origin of the isolates. However, this level of resolution is not sufficient for microepidemiologic analyses. We hypothesized that, as for *M. tuberculosis* (*17*), deletional and insertional events mediated by repetitive sequence elements are a major mechanism for genomic variation in *M. ulcerans*. To test this hypothesis, we developed a plasmid-based microarray and analyzed genomic DNA from 30 *M. ulcerans* isolates of diverse origins.

## Materials and Methods

### Plasmid-Based DNA Microarray

From a shotgun clone library of strain Agy99, 352 *Escherichia coli* plasmids (pCDNA2.1, Invitrogen, Basel, Switzerland) were randomly selected. Each plasmid contained an *M. ulcerans* DNA fragment of ≈2.3–2.7 kb. Given a genome size of 5,806 kb (*18*), this set of plasmid inserts represents a theoretical genome coverage of ≈10%. Plasmid DNA was prepared by using a Biomek 2000 Workstation (Beckman Coulter, Krefeld, Germany) and dissolved at a concentration of 150 ng/µL in 3× SSC (20× SSC stock solution is 3 M sodium chloride, 0.2 M sodium citrate, pH 7.0). The DNA samples were loaded on a piezo-dispensing head that contained 24 channels and spotted onto glass slides coated with poly-L-lysine (Superfrost Plus, Menzel, Braunschweig, Germany) by using a Topspot spotter (Biofluidix, Freiburg, Germany). Slides were incubated at 4ºC overnight and rehydrated under 50%–60% humidity for 1 h at room temperature. The spots resulting from a volume of ≈1 nL had an average diameter of 270 µm and were 500 µm apart from each other. The microarray layout displayed 2 identical fields—for hybridization with 2 different probes—that consisted of 2 replicates each, both of which contained 32 controls and 352 plasmids.

### Biotinylation of *M. ulcerans* Genomic DNA Fragments

**Figure 1:**
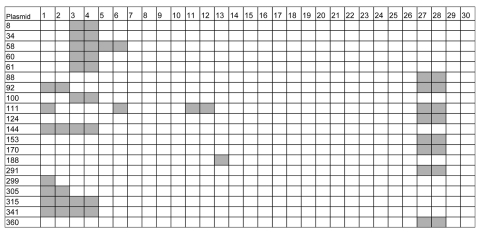
Distribution of outlier signals among the 30 *Mycobacterium ulcerans* isolates tested. *M. ulcerans* isolates: 1, 8756 Japan; 2, 980912 China; 3, 842 Suriname; 4, 7922 French Guiana; 5, 5147 Australia; 6, 5142 Australia; 7, 9549 Australia; 8, 9550 Australia; 9, 940339 Australia; 10, 8849 Australia; 11, 5151 Democratic Republic of Congo (DRC); 12, 5150 DRC; 13, 940511 Côte d'Ivoire; 14, 940662 Côte d'Ivoire; 15, 940815 Côte d'Ivoire; 16, 960658 Angola; 17, 960657 Angola; 18, 970680 Togo; 19, 970321 Ghana; 20, 970483 Ghana; 21, 970359 Ghana; 22, 940111 Benin; 23, 001441 Benin; 24, 940886 Benin; 25, 970104 Benin; 26, 940512 Benin; 27, 5143 Mexico; 28, 5114 Mexico; 29, 941331 Papua New Guinea (PNG); 30, 9537 PNG.

*M. ulcerans* clinical isolates used in this study are listed in Figure 1. Bacterial pellets of about 60 mg (wet weight) were heat inactivated for 1 h at 95°C in 500 µL extraction buffer (50 mmol/L Tris-HCl, 25 mmol/L EDTA, 5% monosodium glutamate) and sequentially treated with lysozyme (2 h, 37°C, 17 M lysozyme) and proteinase K (overnight, 45°C, 0.3 M proteinase K in proteinase K buffer: 1 mmol/L Tris-HCl, 5 mmol/L EDTA, 0.05% sodium dodecyl sulfate [SDS], pH 7.8). After digestion the samples were subjected to bead beater treatment (Mikro-Dismembrator, Braun Biotech International, Berlin, Germany) with 300 µL of 0.1-mm zirconia beads (BioSpec Products, Bartlesville, OK, USA) for 7 min at 3,000 rpm. DNA was extracted from the supernatants by phenol-chloroform (Fluka, Buchs, Switzerland) extraction and ethanol precipitation. Seven micrograms of *M. ulcerans* genomic DNA was digested with 3 U of *Sau*3A1 (New England Biolabs, Hitchin, UK) for 2 h at 37ºC and biotinylated according to Pollack et al. (*19*) using a BioPrime kit (Gibco/BRL, Gaithersburg, MD, USA). The biotinylated DNA was purified by using a Microcone YM-30 filter (Amicon/Millipore, Bedford, MA, USA), and its concentration was measured by optical density at 260 nm (GeneQuant spectrophotometer, Cambridge, UK).

### Hybridization of Microarray Slides

Five micrograms of biotinylated DNA was mixed with 30 µg human Cot-1 DNA (Roche Applied Science, Indianapolis, IN, USA) and 100 µg yeast tRNA (Gibco/BRL). The hybridization mix was concentrated with a Speed Vac Concentrator System (Eppendorf, Basel, Switzerland), resolved in 3× SSC, 0.3% SDS, denatured for 3 min at 95ºC, and incubated for 30 min at 37ºC before hybridization. Microarray slides were cleaned with a nitrogen flow, exposed to UV light in a Stratalinker 2400 (Stratagene, La Jolla, CA, USA) at 650× 100 µJ, and heated for 5 min to 95ºC before application of 13 µL of the hybridization mix on each array field. Hybridization occurred for 20 h at 65ºC in a hydration chamber. Hybridized slides were washed once with 2× SSC, 0.03% SDS for 5 min at 65ºC, twice with 1× SSC for 5 min at room temperature, and finally with 0.2× SSC for 5 min at room temperature. The coloration step was performed with 2 mL staining solution containing 50% caseine, 1× maleic acid buffer (Roche Applied Science), and 2 µg Streptavidin Cy3 Fluorolink (Amersham, Piscataway, NJ, USA) for 30 min at room temperature, followed by additional washings for 5 min with 1× TBS (0.15 M sodium chloride, 0.02 M Tris, pH 7.5) as well as 0.1× TBS and drying with a nitrogen flow. DNA of all 30 *M. ulcerans* strains was processed under identical conditions and hybridized at least twice, which yielded 4 sets of data for each strain. Human Cot-1 DNA and plasmid DNA without insert as well as a hybridization mix without DNA served as negative controls for hybridization. A 500-bp β-lactamase gene fragment and Cy3-labeled random oligonucleotides (Microsynth, Balgach, Switzerland) were used as positive controls and for estimation of the amount of spotted DNA.

### Microarray Scanning and Data Evaluation

Images of the microarrays were acquired by using a laser microarray scanner (GenePix 4100A, Axon Instruments Inc., Foster City, CA, USA) with an excitation wave length of 532 nm, an emission wavelength of 570 nm, and standardized measurement parameters. The resulting image was analyzed by the software GenePix Pro 4.1 (Axon Instruments Inc.), which enabled assignments of mean intensity values used for data interpretation. To select spots to be included in the analysis of genomic diversity of *M. ulcerans* strains, replicates of 10 hybridizations were performed by using *M. ulcerans* Agy99 genomic DNA. All spots that showed a signal lower than twice that given by the negative control plasmid without insert were rejected, as were all spots for which coefficient of variation was >30%. Further analysis used 232 spots that had an average signal above the threshold and sufficient signal stability. For each plasmid, we calculated the average signal value, standard deviation, and coefficient of variation and assessed a signal ratio in comparison with the reference strain. Outlier spots with a ratio higher than U2 (U2 = upper quartile + 3× interquartile) were identified through a box-plot analysis.

### Characterization of Large Sequence Polymorphisms

Microarray data that indicated the presence of a deletion were verified by PCR analysis, which used primer pairs that spanned the insertion sequences of the respective plasmids, the flanking regions, or both. The 5′ and 3′ limits of the confirmed genomic deletions with respect to the genome of strain Agy99 were determined by PCR analysis, which used multiple sets of primers complementary to flanking genomic regions. PCR analyses that bridged the genomic breakpoints were performed by using a long-range PCR polymerase mix (Fermentas, St Leon-Rot, Germany) according to the manufacturer’s description. PCR products were cloned into pGEM-T (Catalys AG, Promega, Wallisellen, Switzerland) and sequenced using an ABI PRISM 310 genetic sequence analyzer (Perkin-Elmer, Waltham, MA, USA).

## Results

### Comparative Genomic Hybridization of *M. ulcerans* Isolates

**Figure 2:**
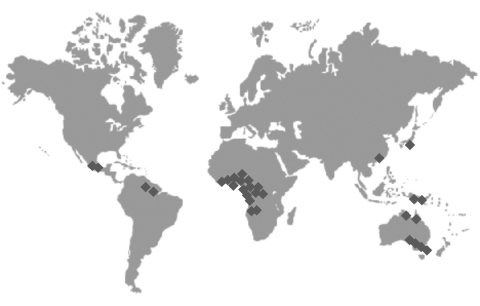
Distribution map of *Mycobacterium ulcerans* patient isolates used in this study (strain identity as listed in Figure 1).

**Figure 3:**
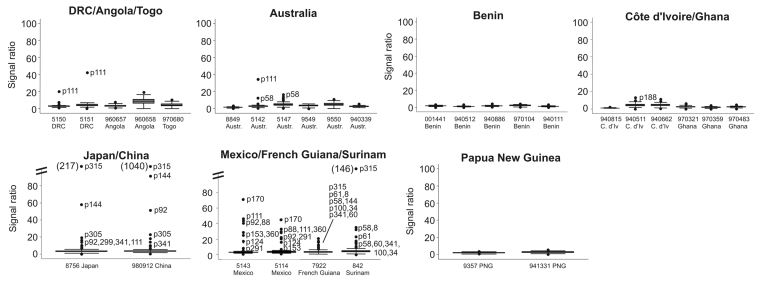
Box-plot analysis of the signal ratios obtained with genomic DNA of 30 *Mycobacterium ulcerans* strains identifying plasmids yielding outlier signals. Shown is the signal ratio in comparison with the African reference strain Agy99. The median of the ratios obtained is represented by the line in the center of the rectangular box. The 2 ends of the rectangles represent the upper quartile (UQ), which corresponds to the 75th percentile, and the lower quartile (LQ), which corresponds to the 25th percentile. The interquartile (IQ) is equal to UQ – LQ. The other 2 values shown are the maximum and minimum value of the data set: U1 = UQ + 1.5IQ; L1 = LQ – 1.5IQ. Double slashes represent discontinued range on the y-axis, values of the outliers beyond 100 in brackets. DRC, Democratic Republic of Congo; Aust., Australia; C. d’Iv, Côte d’Ivoire. PNG, Papua New Guinea.

We constructed a microarray based on a random selection of 232 *Escherichia coli* plasmids obtained from a shotgun sequence library of the *M. ulcerans* isolate Agy99 from Ghana. Genomic DNA hybridization signal intensities from 30 *M. ulcerans* clinical isolates of worldwide distribution (Figure 2) were compared with those obtained with strain Agy99. Box-plot analysis (Figure 3) identified plasmids that yielded outlier signals with respect to strain Agy99. For 19 of 20 plasmids, PCR analysis confirmed an association of the outlier signal with a genomic deletion. Only 1 low hybridization signal represented a false-positive result (p188 from strain 940511, Côte d’Ivoire; Figure 3). The number of confirmed outlier plasmids per isolate ranged from zero for most African isolates to 9 for isolates from Suriname and French Guiana (Figure 1).

**Figure 4:**
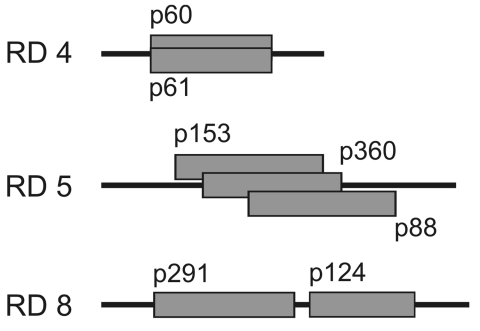
Positions of overlapping or adjacent plasmid inserts in regions of difference (RDs) 4, 5, and 8. Identical results retrieved by different plasmids with overlapping sequences or sequences in closest proximity demonstrate the reproducibility and reliability of the differential genomic hybridization method.

Of the 19 plasmid inserts that yielded confirmed outlier signals, 3 (p111, p299, and p341) contained sequences from the virulence plasmid pMUM001 of *M. ulcerans*. Of the 16 plasmids derived from the *M. ulcerans* chromosome, some contained fragments that overlapped the same region (Figure 4). Hybridizing regions were almost identical for p60 and p61. Both plasmids yielded outlier values with the isolates from Suriname and French Guiana. A cluster of overlapping inserts was observed for p88, p153, and p360; these produced outlier values for both of the Mexican isolates. The same pattern was seen with p124 and p291, which have inserts that are located in close proximity to each other in the genome (Figure 3). These results from related inserts demonstrated the reproducibility of the differential hybridization analysis. Because the inserts p60–p61, p88–p153–p360, and p124–p291 were part of the same deletion in regions of difference (RDs) 4, 5, and 8, respectively; (Figure 4), altogether 12 chromosomal RDs were identified.

### Characterization of Genomic RDs

The 5′ and 3′ limits of the genomic deletions with respect to the genome of strain Agy99 were determined by PCR analysis that used multiple sets of primers complementary to plasmid inserts and to flanking genomic regions. The size of the deletions ranged from 1.8 kb to 53.1 kb (Table).

**Table:** Features of the 15 distinct deletions identified in *Mycobacterium ulcerans* isolates*

RD	Deletion no.	Plasmid no.	Origin	Size of deletion, kb	No. CDSs and pseudogenes
1	1	p8	SU/FG	11.1	14
2	2	p34	SU/FG	6.6–6.8	4
3	3A	p58	AU1/AU2	3.5	5
	3B	p58	SU/FG	3.8	6
4	4	p60, p61	SU/FG	1.8–2.4	2
5	5	p88, p153, p360	ME1/ME2	27.1–27.4	23
6	6	p92	JP/CH/ME1/ME2	19.7	24
7	7	p100	SU/FG	15.3	13
8	8	p124, p291	ME1/ME2	52.8–53.1	50
9	9A	p144	JP/CH	18.1	15
	9B	p144	SU/FG	25.4	20
10	10	p170	ME1/ME2	8.2–8.7	10
11	11	p305	JP/CH	4.6	7
12	12A	p315	JP/CH	53.1	50
	12B	p315	SU/FG	35.4–35.5	32

*RD, region of difference; CDSs, coding sequences; SU, Suriname; FG, French Guiana; AU, Australia; ME, Mexico; JP, Japan; CH, China.

In 3 of the 12 RDs (RD3, 9, and 12), 2 distinct types of overlapping deletions (designated A and B) were observed, leading to a total of 15 large deletions. The overlapping deletions shared neither common 5′ nor 3′ end sequences. The strains from Australia had a 3.5-kb deletion in RD3; strains from Suriname and French Guiana had a slightly larger (3.8-kb) deletion. The isolates from Suriname and French Guiana had a larger (25.4-kb) deletion in RD9 than the isolates from Japan and China (17.7 kb). The largest deletion (53.1 kb) was designated RD12A and was observed in strains from Japan and China. Isolates from Suriname and French Guiana had a significantly smaller deletion in RD12 (35.2 kb). The 19.7-kb deletion 6 was found in isolates from 2 different regions (Mexico and Japan/China, respectively). All other deletions were observed in 2 isolates from the same region (Table).

To assess whether polymorphisms undetected by the microarray analysis would frequently occur in the identified RDs, we performed a detailed PCR analysis in all 30 *M. ulcerans* strains included in this study for 2 randomly selected RDs (RD5 and 12). We used 4 distinct primer pairs to span the insert sequence plus 5′ and 3′ flanking sequence stretches. For RD12, the PCR analysis confirmed the presence of a deletion in the 4 strains that had outlier signals in the microarray analysis, but no evidence for deletional polymorphism was obtained in the other strains. For RD5, PCR analysis confirmed the presence of a deletion in the 2 Mexican strains that had outlier signals (not shown). In addition, this PCR analysis identified the presence of an insertion in strains from Japan, China, Suriname, and French Guiana. The sequence of this 765-bp DNA insert was identical for all 4 strains. Its G+C content was 64%, and BLAST searches showed 98% identity with a sequence stretch of the *M. marinum* genome (www.sanger.ac.uk/cgi-bin/blast/submitblast/m_marinum) but no significant homology with sequences in the National Center for Biotechnology Information BLAST databases (www.ncbi.nlm.nih.gov/blast).

### Association of Deletions with Insertions

**Figure 5:**
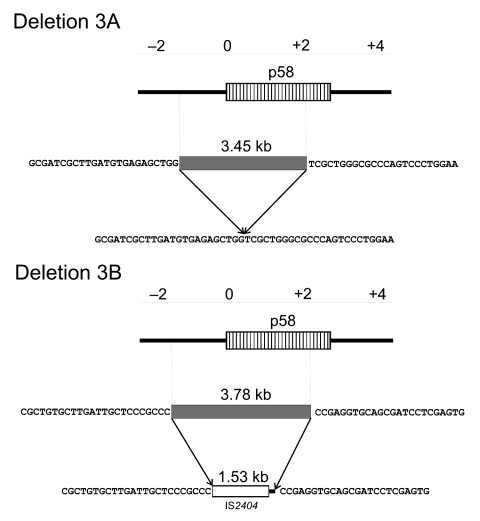
Two distinct deletions in region of difference 3. Although deletion 3A represents a mere deletion event, the larger deletion 3B is associated with an insertion event. Neither 5’ nor 3’ ends are identical in the 2 deletions.

Of the 15 identified genome rearrangement events, 1 (deletion 3A observed in 2 Australian isolates) was found to be a deletion, with the genomic sequences flanking the 5′ and 3′ borders of the 3,451-bp deletion being directly joined (Figure 5). Analysis of the other 14 deletions showed that the loss of DNA in a given strain with respect to the genome of Agy99 was associated with the insertion of substituting sequences of varying sizes unrelated to the deleted regions. As an example, the larger (3,784-bp) deletion 3B found in the isolates from Suriname and French Guiana was associated with the insertion of an unrelated DNA fragment, which comprised the 1,368 bp of IS*2404* (*20*) plus an additional DNA stretch of 163 bp (Figure 5). For most of the other deletions, 1 of the 2 highly abundant insertion sequence elements (IS*2404* or IS*2606*) was situated in either the genomic sequences that flanked the deletion or that were in the deleted parts or in the substituting sequence stretches (as for deletion 3B).

### Analysis of Coding Sequences and Pseudogenes in the Deleted DNA Sequences

**Figure 6:**
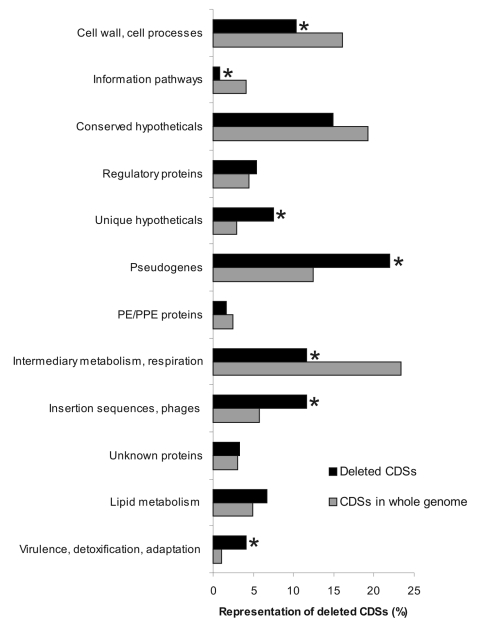
Functional categories of coding sequences (CDSs) and pseudogenes and their frequencies in the deletions and the whole genome. CDSs and pseudogenes in the identified regions of difference (RDs) and in the entire reference *Mycobacterium ulcerans* Agy99 genome were functionally categorized; the frequencies of individual categories are compared. Statistically overrepresented in the RDs were pseudogenes; insertion sequences; CDSs encoding unique hypothetical proteins; and proteins involved in virulence, detoxification, and adaptation. *Significant differences (p<0.05).

The 15 deletions identified contained 52 pseudogenes and 185 predicted protein-coding sequences (CDSs), which represent 5.7% of the annotated 4,143 CDSs in the genome of the *M. ulcerans* strain Agy99 (*18*). The number of deleted CDSs and pseudogenes ranged from 2 (RD4) to 50 (RD8 and 12A) and averaged 18.6 per deletion (Table). CDSs were classified into 11 functional categories (*17*). When compared with the gene composition of the entire Agy99 genome, the following functional categories were overrepresented among the 185 deleted CDSs: insertion sequences, unique hypothetical genes, and predicted proteins involved in detoxification (Figure 6). Also overrepresented was the deletion of the 52 pseudogenes that contain frame shift mutations and premature stop codons or that are disrupted by an insertion sequence. In contrast, genes involved in intermediary metabolism, information pathways, and cell wall/cell processes were underrepresented among the deleted CDSs (Figure 6). Of the 185 deleted functional CDSs, 89 had orthologs with >50% amino acid sequence identity to proteins from the *M. tuberculosis* H37Rv genome. A tendency for gene categories to cluster within the RDs was found. RD2 comprises 2 PPE genes: RDs 1, 12A, and 12B are predominantly CDSs involved in lipid metabolism, and RDs 9A and 11 include mainly transcriptional regulators. However, overall *M. ulcerans* lineages from distinct geographic origin (Africa, Australia, Asia, South America, Mexico) did not differ markedly in the categories of deleted genes. RD8 (deleted in the Mexican strains) is particularly interesting because it contains a cluster of proteins of the mammalian cell entry *mce3* operon and associated regulators thereof. The transcriptional repressor, *Mce3R*, is considered to be an essential gene required for growth of *M. tuberculosis* (*21*). In addition, RD8 comprises a collection of CDSs of almost every functional category (Appendix Table). The spectrum of RD8-associated CDSs involved in detoxification included the multidrug transport protein *mmr*, the epoxide hydrolase *EphB*, the thiol peroxidase *Tpx*, and the alkyl hydroperoxide reductase C protein *AhpC*.

Although CDSs involved in intermediary metabolism were underrepresented among the deleted genes, 21 (42%) of deleted CDSs of this category were dehydrogenases (such as acyl-CoA short-chain alcohol, saccharopine, and aldehyde dehydrogenases), which are central enzymes in anaerobic metabolism (*22*) and important for survival in poorly oxygenated environments such as soil (*23*). In addition, other genes associated with anaerobic respiration, such as nitroreductases and electron transfer proteins, were found among the deleted CDSs.

## Discussion

We describe the use of a plasmid-based DNA microarray for identifying large deletional and insertional genomic polymorphisms in a collection of 30 *M. ulcerans* strains of geographically diverse origin. A set of plasmids randomly selected from an *E. coli* shotgun library of *M. ulcerans* genomic DNA was spotted on microarray slides. This is a newly developed technology, highly suitable for situations in which the complete genome sequence of a microorganism is not available. The prototype array used comprised 232 plasmids that yielded a reproducible and stable signal. Plasmids contained *M. ulcerans* genomic DNA fragments of 2.3–2.7 kb, thus reaching a theoretical genome coverage of 10%. Despite this incomplete coverage, 12 chromosomal and 3 virulence plasmid–associated RDs were identified. Fifteen distinct deletions of 1.8–53.1 kb were found and characterized in detail by sequence analysis within the 12 genomic RDs. The deletions identified were found in >1 *M. ulcerans* isolate, which demonstrates that they do not reflect events that occur during in vitro cultivation of individual isolates*.* The diversity of deletions within some genomic regions implies recombination hot spots or a selective advantage for loss of particular sequence stretches. Recombination events between adjacent copies of IS*6110* in *M. tuberculosis* and IS*100* in *Yersinia pestis* have been shown to promote the deletion of intervening DNA segments (*9*,*23*–*26*). Close association of RDs with the high copy number elements IS*2404* and IS*2606* of *M. ulcerans* indicates that these are involved in insertional and deletional events.

Although genome coverage with the prototype microarray used here was low, several geographic types of *M. ulcerans* could be differentiated. The largest group comprised all the African isolates (from Ghana, Benin, Côte d’Ivoire, Democratic Republic of Congo, Angola, and Togo), the isolates from Papua New Guinea, and some of the Australian isolates. A second group comprised the Australian strains 5142 and 5147, and a third group included the South American strains (from Suriname and French Guiana). The Mexican isolates represented a fourth; the Asian isolates (from Japan and China), a fifth subgroup. An extended analysis of insertions and deletions is expected to eventually give insight into the phylogenetic relationship between *M. marinum* and different lineages of *M. ulcerans*. Moreover, the use of a microarray that covers the whole genome may lead to the development of a genomic fingerprinting method, which is urgently needed for microepidemiologic studies that aim to characterize transmission pathways and environmental reservoirs of *M. ulcerans*.

The 15 distinct genomic deletions that we identified affected 6.2% of the *M. ulcerans* Agy99 genome, or 5.7% of the annotated CDSs and pseudogenes. When a whole-genome microarray was used to compare genomic DNA of 100 *M. tuberculosis* isolates, 5.5% of the genes were found to be affected (*27*). When one considers the limited genome coverage of the *M. ulcerans* prototype array used here, findings demonstrate a remarkably high degree of insertional and deletional diversity in *M. ulcerans.* In contrast, single nucleotide polymorphisms are rare (*14*).

Comparative genomic studies have shown that *M. ulcerans* recently evolved from the ubiquitous, fast-growing environmental bacterium *M. marinum* (www.sanger.ac.uk/projects/m_marinum) by lateral gene transfer and reductive evolution (*18*). Our comparative genomic hybridization analysis of a worldwide collection of *M. ulcerans* strains indicates that the downsizing of the genome from 6.6 Mb (*M. marinum*) to 5.8 Mb (*M. ulcerans* Agy99) is an ongoing process. Further genome reduction appears to be driving genetic diversification of *M. ulcerans.* Studies of other groups of microorganisms indicate that genome reduction is usually associated with adaptation to a more stable environment. An example is *M. leprae,* which has eliminated >2,000 genes upon adaptation to its human host (*28*). To which ecologic niche(s) in the environment or in host organisms *M. ulcerans* is adapting remains to be investigated.

Among the deleted CDSs are 11 members of the mammalian cell entry *mce3* operon, which are regarded as virulence determinants in other mycobacteria. In *M. tuberculosis* the *mce* operons have been shown to code for genes important for entry and survival of the pathogen in mammalian cells (*29*,*30*). The 4 *mce* operons of *M. tuberculosis* have homologs among other mycobacteria. In particular, the *mce3* operon has been found in *M. avium* and *M. smegmatis;* its deletion in *M. bovis* has been also documented (*31*). The 12.7-kb region that codes for the *mce3* operon is located near the 3′ end of the RD2 element (*32*) that is present in *M. bovis* but absent in some strains of *M. bovis* BCG, which suggests the potential instability of this region. A mouse model of intradermal infection has recently shown that *M. ulcerans* is initially captured by phagocytes (*33*). In vitro studies suggest that the *M. ulcerans* intracellular stage is transient because phagocytic cells enter apoptosis-mediated cell death within 1 day. It will be interesting to investigate whether the *mce3* operon plays a role during the transient invasion of host cells by *M. ulcerans.*

Overrepresentation of proteins involved in detoxification processes among the deleted CDSs indicates adaptation to a more stable environment. Deletion of many dehydrogenases thought to be involved in anaerobic respiration and of anaerobic respiratory enzymes and tranporters may give a hint that this niche is not anaerobic. At least in highly disease-endemic areas, *M. ulcerans’* long-term persistence in chronic wounds and shedding into the environment may be relevant for the propagation of this species. Whether *M. ulcerans* is primarily adapting to persist in a specialized environmental habitat, in arthropod hosts (*34*), or in chronic wounds of mammalian hosts remains to be determined.

## Supplementary Material

Appendix Table Deleted coding sequences in RD8 (Mexican strains) of Mycobacterium ulcerans*
