# Supplementary material for: Ongoing Genome Reduction in Mycobacterium ulcerans
Source: Emerg Infect Dis. 2007 Jul;13(7):1008–15. doi: 10.3201/eid1307.060205 (PMC2878211; doi:10.3201/eid1307.060205)
Supplement: Appendix Table — Deleted coding sequences in RD8 (Mexican strains) of Mycobacterium ulcerans* [file 06-0205_appT-s1.pdf]

Appendix Table. Deleted coding sequences in RD8 (Mexican strains) of *Mycobacterium ulcerans**

| Deleted coding sequences | Functional classification | Description |
|---|---|---|
| MUL_2863 | Cell wall/cell processes | Conserved mce associated membrane protein |
| MUL_2864 | Cell wall/cell processes | Conserved mce associated membrane protein |
| Mce3F | Cell wall/cell processes | Mce-family protein Mce3F |
| lprM | Cell wall/cell processes | Mce-family lipoprotein LprM |
| mce3D | Cell wall/cell processes | Mce-family protein Mce3D |
| mce3C | Cell wall/cell processes | Mce-family protein Mce3C |
| mce3B | Cell wall/cell processes | Mce-family protein Mce3B |
| mce3A | Cell wall/cell processes | Mce-family protein Mce3A |
| yrbE3B | Cell wall/cell processes | Conserved hypothetical integral membrane protein, YrbE family, probably part of mce3 operon |
| yrbE3A | Cell wall/cell processes | Conserved hypothetical integral membrane protein YrbE3A |
| MUL_2873 | Intermediary metabolism | Ferredoxin, Fe-S protein involved in electron transfer |
| MUL_2874 | Conserved hypothetical | Conserved hypothetical protein, has domain identity with ketosteroid isomerase-related proteins |
| Cyp278A1P | Pseudogene | Cytochrome P450, heme-thiolate monooxygenase |
| MUL_2876 | Regulatory protein | Conserved hypothetical regulatory protein, has weak domain identity with 2-component response regulators |
| MUL_2877 | Intermediary metabolism | Aldehyde dehydrogenase, probably oxidizing aliphatic and aromatic aldehydes |
| MUL_2878 | Conserved hypothetical | Conserved hypothetical protein |
| mce3R | Regulatory protein | Transcriptional repressor Mce3R, probably TetR-family, repressor of the mce3 operon, probably also has regulatory action on the mce2 operon |
| MUL_2881 | Detoxification | Multidrug transport integral membrane protein Mmr, export of multidrug substrates leading to multidrug resistance |
| MUL_2882 | Conserved hypothetical | Conserved hypothetical protein |
| MUL_2884 | Conserved hypothetical | Conserved hypothetical membrane protein |
| MUL_2885 | Intermediary metabolism | Dehydrogenase/reductase, short-chain type |
| ribA1 | Intermediary metabolism | Riboflavin biosynthesis protein, RibA1 |
| MUL_2887 | Intermediary metabolism | Conserved hypothetical oxidoreductase |
| ephB | Detoxification | Epoxide hydrolase, EphB, acts on epoxides (alkene oxides, oxiranes), involved in xenobiotic detoxification, determines steady-state levels of physiological mediators |
| MUL_2889 | Intermediary metabolism | Oxygenase, probably involved in electron transfer |
| MUL_2890 | Intermediary metabolism | Oxygenase |
| echA13 | Pseudogene | Enoyl-CoA hydratase, EchA13, probably involved in oxidization of fatty acids |
| fadE17 | Lipid metabolism | Acyl-CoA dehydrogenase FadE17, probably involved in lipid degradation |
| fadE18 | Lipid metabolism | Acyl-CoA dehydrogenase FadE18, probably involved in lipid degradation |
| Tpx | Detoxification | Thiol peroxidase, Tpx, has antioxidant activity, probably removes peroxides |
| MUL_2895 | Intermediary metabolism | Conserved hypothetical oxidoreductase, domain identity with Fe-S oxidoreductases |
| MUL_2896 | IS, prophages | Transposase for IS*2404* |
| MUL_2897 | Pseudogene | Conserved hypothetical protein |
| MUL_2898 | IS, prophages | Transposase for IS*2606* |
| MUL_2900 | Pseudogene | Short-chain fatty acid transporter |
| MUL_2901 | IS, prophages | Transposase for IS*2404* |
| MUL_2902 | Regulatory protein | Transcriptional regulatory protein |
| MUL_2903 | Pseudogene | Conserved hypothetical protein |
| MUL_2904 | Conserved hypothetical | Conserved hypothetical membrane protein |
| MUL_2905 | Intermediary metabolism | Conserved hypothetical protein, contains dihydrofolate reductase domain |
| MUL_2906 | Conserved hypothetical | Conserved hypothetical protein, has weak identity with hydrolase domains |
| MUL_2907 | Conserved hypothetical | Conserved hypothetical protein |
| MUL_2908 | Pseudogene | Dehydrogenase/reductase, short-chain type |
| MUL_2909 | Conserved hypothetical | Conserved hypothetical protein |
| MUL_2910 | Unique hypothetical | Unique hypothetical protein |
| MUL_2911 | Intermediary metabolism | Alpha-L-fucosidase, involved in carbohydrate transport and metabolism |
| ahpC_1 | Detoxification | Alkyl hydroperoxide reductase C protein, AhpC, involved in oxidative stress response |
| MUL_2913 | IS, prophages | Transposase for IS*2606* |
| MUL_2914 | Intermediary metabolism | Metal-dependent hydrolase |
| MUL_2915 | IS, prophages | Transposase for IS*2404* |

*RD, regions of difference; IS, insertion sequence. Gray shading indicates categories for which a biological function can be discussed.
